# Health-Related Concerns of Anti-LGBTQ+ Legislation: Thematic Analysis Using Social Media Data

**DOI:** 10.2196/68956

**Published:** 2025-09-11

**Authors:** Ari Z Klein, Kaelen Spiegel, José A Bauermeister, Graciela Gonzalez-Hernandez

**Affiliations:** 1 Department of Biostatistics, Epidemiology, and Informatics Perelman School of Medicine University of Pennsylvania Philadelphia, PA United States; 2 Department of Computational Biomedicine Cedars-Sinai Medical Center West Hollywood, CA United States; 3 Department of Family and Community Health School of Nursing University of Pennsylvania Philadelphia, PA United States

**Keywords:** LGBTQ+, lesbian, gay, bisexual, transgender, queer/questioning, sexual and gender minorities, legislation, policy, social media, natural language processing

## Abstract

**Background:**

There has been a recent proliferation of anti-LGBTQ+ (lesbian, gay, bisexual, transgender, queer/questioning) legislation being proposed in the United States, including more than 500 bills across 42 states in 2024. Many of the studies examining the impact of anti-LGBTQ+ legislation have focused specifically on the association with mental health outcomes.

**Objective:**

The objective of this study was to use social media data to more broadly explore health-related concerns of anti-LGBTQ+ legislation among sexual minority men in the United States.

**Methods:**

We leveraged a dataset containing 70 million tweets that were posted by 23,276 users in the United States who self-reported on Twitter that they are sexual minority men. First, we searched these tweets for keywords related to LGBTQ+ legislation. Next, we developed a codebook for identifying those that expressed health-related concerns of anti-LGBTQ+ legislation. Then, we developed a coding scheme to categorize these concerns into one or more themes by using an inductive approach. Finally, we automatically identified the users’ geographic location and age for subgroup analyses.

**Results:**

Among 8486 keyword-matched tweets, 493 (5.8%) tweets expressed health-related concerns due to anti-LGBTQ+ legislation and were posted by 288 sexual minority men in the United States: 112 (38.9%) who posted about *health care*, 84 (29.2%) about *safety*, 64 (22.2%) about *mental health*, 62 (21.5%) about *general harm*, 49 (17%) about *human rights*, and 40 (13.9%) about *support*. *Health care* was the top concern overall and across the United States and age groups. In contrast, the higher prevalence of *mental health* was driven by the larger number of users in the South, as it was less of a concern in other regions. Similarly, *mental health* was less of a concern among older age groups. *Safety* was as much of a concern as *mental health* overall and across the United States and most age groups.

**Conclusions:**

Our findings may inform a broader range of health interventions and approaches for targeting them at specific populations of sexual minority men. By demonstrating that these concerns are expressed on social media, our findings can be leveraged by advocacy groups to amplify voices and rally public support for countering anti-LGBTQ+ bills.

## Introduction

There has been a recent proliferation in anti-LGBTQ+ (lesbian, gay, bisexual, transgender, queer/questioning) legislation being proposed in the United States, including more than 500 bills across 42 states in 2024 [1]. Many bills target youth, including 78 bills that would age-restrict gender-affirming health care and 209 that would restrict schools. For example, Florida’s Parental Rights in Education Act (HB 1557), commonly referred to by critics as the “Don’t Say Gay” law, was passed in 2022 and has prompted other states to introduce similar legislation that prohibits public schools from instruction on sexual orientation or gender identity and from withholding confidential disclosures from students’ parents. Many of the studies examining the impact of anti-LGBTQ+ legislation have focused on mental health [2-5]. Other studies that have taken more exploratory approaches using thematic analysis have been limited to a specific age group (85.8% aged ≤21 years) [6] or geographic location (one state) [7]. Although social media has been used to explore the impact of equitable LGBTQ+ legislation [8], it has not been used to study anti-LGBTQ+ legislation. The objective of this study was to use social media data for a thematic analysis that more broadly explores health-related concerns of anti-LGBTQ+ legislation among sexual minority men, including by geographic location and age.

## Methods

### Ethical Considerations

The institutional review boards of the University of Pennsylvania (828972) and Cedars-Sinai Medical Center (STUDY00002429) deemed this study exempt. The data used in this study were publicly available at the time of collection and were collected and analyzed in accordance with the Twitter terms of service. We have slightly paraphrased the sample tweets to deidentify the users.

### Study Population and Data Collection

We leveraged a dataset from our prior work containing 70 million tweets that were posted by 23,276 users in the United States who self-reported on Twitter that they are sexual minority men [9]. We searched these tweets for *parental rights in education* or *don’t say gay*, including in hashtags, or keywords related to both legislation (*legislation*, *bill*, *bills*, *law*, *laws*, *policy*, *policies*, *ban*, *bans*, *banning*, *proposal, proposals*) and LGBTQ+ (*lgbt*, *lgbtq*, *gay*, *bisexual*, *trans*, *transgender*, *queer*, *sex*, *gender*, *saygay*). Our query returned 8486 tweets, excluding retweets, posted by 2857 users between September 2009 and September 2022.

### Thematic Analysis

We used 200 of the 8486 tweets—1 random tweet per user—to develop a codebook for identifying those that expressed health-related concerns of anti-LGBTQ+ legislation (Multimedia Appendix 1). We adopted the World Health Organization’s (WHO’s) broad definition of *health* as “a state of complete physical, mental and social well-being” [10]. We included nonpersonal experiences but excluded references to federal, international, or equitable legislation. To assess interannotator agreement, 2 authors (AZK and KS) coded an additional 200 tweets. After resolving disagreements, one of the authors (KS) labeled the remaining 8086 tweets.

We used 50 tweets to develop a coding scheme for categorizing the health-related concerns into one or more themes. In contrast to a *deductive* (theory-driven) approach, we used an *inductive* (data-driven) approach to thematic analysis and focused more on themes that captured explicit, rather than latent, patterns of meaning [11]. To assess interannotator agreement, 2 authors (AZK and KS) coded an additional 50 tweets. After resolving disagreements, one author (KS) coded the remaining tweets. For subgroup analysis, we used automated tools to identify the users’ geographic location based on the metadata of their tweets and user profiles (Carmen) [12] and age based on explicit self-reports in their tweets (ReportAGE) [13].

## Results

Among 8486 keyword-matched tweets, we determined that 614 (7.2%) tweets, posted by 339 users, expressed health-related concerns of anti-LGBTQ+ legislation. Interannotator agreement was 96%, and Cohen κ was 0.80 [14]. Among the 339 users who had been automatically identified [9], we manually validated that 288 (85%) self-reported being sexual minority men (Table 1). Among the 614 tweets, 493 (80.2%) were posted by 288 validated users.

**Table 1:** Sample tweets posted by a single Twitter user that illustrate true positive (tweet 1) and false positive (tweet 2) self-reports of being a sexual minority man and true positive (tweet 3) and false positive (tweet 4) self-reports of age. The timestamps indicate that this user was aged 51 years at the time of expressing a health-related concern of anti-LGBTQ+ (lesbian, gay, bisexual, transgender, queer/questioning) legislation (tweet 5).

	Tweet	Timestamp
1	As a gay man coming of age in the 80s, I often think about what the world would be like if society had focused on HIV as much it has on COVID. These vaccines are miraculous.	January 15, 2021
2	Coming out as a pro athlete, especially in the NFL, is not easy. I thank Carl Nassib for his courage to be his authentic self as a gay man, and I look forward to him signing with a new team.	March 16, 2022
3	I turned 50 today. Turning 50 during a pandemic isn’t exactly what I had planned, but life happens. My friends shipped me a birthday cake, which I’ll eat as we celebrate on Zoom.	May 11, 2020
4	Passing Prop E is probably the worst political failure I have seen in my 23 years of living in San Francisco. It will reduce funding for affordable housing/transit and push out start-ups/nonprofits.	March 4, 2020
5	Alabama’s anti-trans law forces students to use birth-assigned-gender restrooms & requires teachers to out students to their parents, putting their safety at risk. It’s hard to overstate how harmful this law is. It will cause the death of trans kids.	May 8, 2022

Among the 493 tweets, we coded 202 (41%) as *health care*, 106 (21.5%) as *general harm*, 99 (20.1%) as *safety*, 77 (15.6%) as *mental health*, 51 (10.3%) as *human rights*, and 43 (8.7%) as *support* (Table 2). Strict interannotator agreement—that is, for all themes per tweet—was 76%, and Cohen κ was 0.70. The two authors agreed on at least one common theme for 94% of the tweets. Among the 288 users, 73 (25.3%) posted multiple tweets; so, users who posted less frequently may be underrepresented. Counting each theme once per user, 112 (38.9%) posted about *health care*, 84 (29.2%) about *safety*, 64 (22.2%) about *mental health*, 62 (21.5%) about *general harm*, 49 (17%) about *human rights*, and 40 (13.9%) about *support* (Table 2).

**Table 2:** Definitions, examples, and numbers of tweet-level and user-level themes of health-related concerns of anti-LGBTQ+ (lesbian, gay, bisexual, transgender, queer/questioning) legislation expressed on Twitter by sexual minority men in the United States.

Theme	Definition	Example tweet	Tweets (n=493), n (%)	Users (n=288), n (%)
Health care	Barriers to health care, such as banning gender-affirming care, allowing providers to refuse care, and denying insurance coverage.	They were forced to move after Arkansas banned gender-affirming care for trans minors. The law would have prevented their sons from taking testosterone.	202 (41)	112 (38.9)
Safety	Threats to safety, such as violence, harassment, hate crimes, and unsafe school and home environments.	Alabama’s anti-trans law forces students to use birth-assigned-gender restrooms and requires teachers to out students to their parents, putting their safety at risk. It’s hard to overstate how harmful this law is. It will cause the death of trans kids.	99 (20.1)	84 (29.2)
Mental health	Negative impacts on mental health, such as depression, anxiety, stress, and suicide.	@[username] received approximately 4000 crisis contacts from transgender and nonbinary youth in Texas alone in 2021. Many directly stated that they feel stressed and are considering suicide as a result of anti-trans laws, including sports bans, being debated in the state.	77 (15.6)	64 (22.2)
Human rights	Violations of human rights, such as unconstitutionality, discrimination, and oppression.	Our leaders in Florida are taking our rights away one by one, day by day. The don't say gay bill just passed another round of votes, which prohibits the word gay and other lgbt terms in schools, and also requires teachers to out their students.	51 (10.3)	49 (17)
Support	Lack of support, such as removing safe spaces from schools, limiting discussion and resources, feeling isolated, and inhibiting identity disclosure.	According to the Mayo Clinic, kids can identify genders by 18 months and can have a sense of their own gender by 3 years old. Discussing gender and love is not the same as discussing sexual acts. Supporting kids is hindered by these laws.	43 (8.7)	40 (13.9)
General harm	Concerns about harm in general, without specifying another theme.	I knew I was gay at 8 years old. This erasure will be harmful in the long term, guaranteed. #DontSayGay	106 (21.5)	62 (21.5)

**Figure 1:**
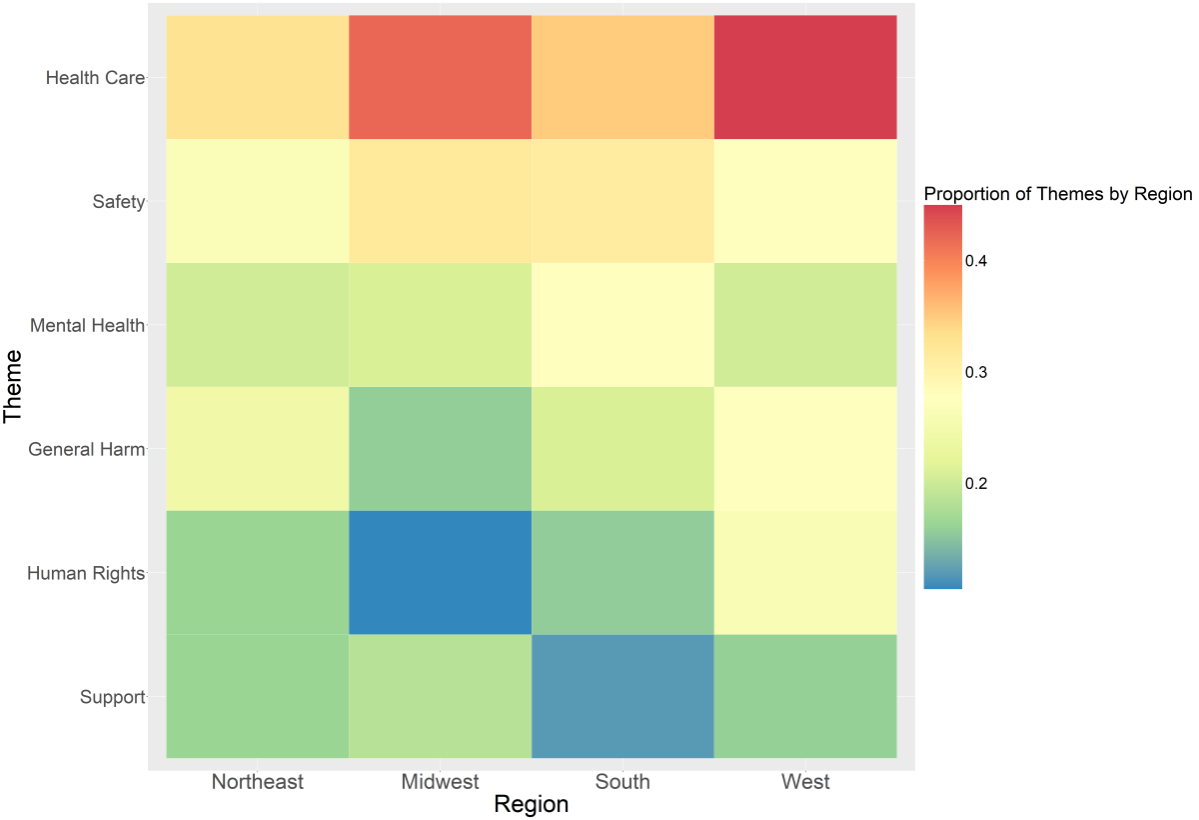
Health-related concerns of anti-LGBTQ+ (lesbian, gay, bisexual, transgender, queer/questioning) legislation expressed by sexual minority men on Twitter, by United States region (n=265).

Carmen [12] identified the geographic location for 265 (92%) of the 288 users, including 36 states: 49 (18.5%) in the Northeast, 38 (14.3%) in the Midwest, 109 (41.1%) in the South, and 69 (26%) in the West [15]. *Health care* and *safety* remained the top concerns across all regions (Figure 1). In contrast, the higher prevalence of *mental health* was driven by the larger number of users in the South (27.5%), as it was lesser than other concerns in the Northeast (20.4%) and West (20.3%). The relatively low prevalence of *support* was also driven by the larger number of users in the South (11.9%), as it was equivalent to or more than other concerns in the Midwest (18.4%) and Northeast (16.3%).

**Figure 2:**
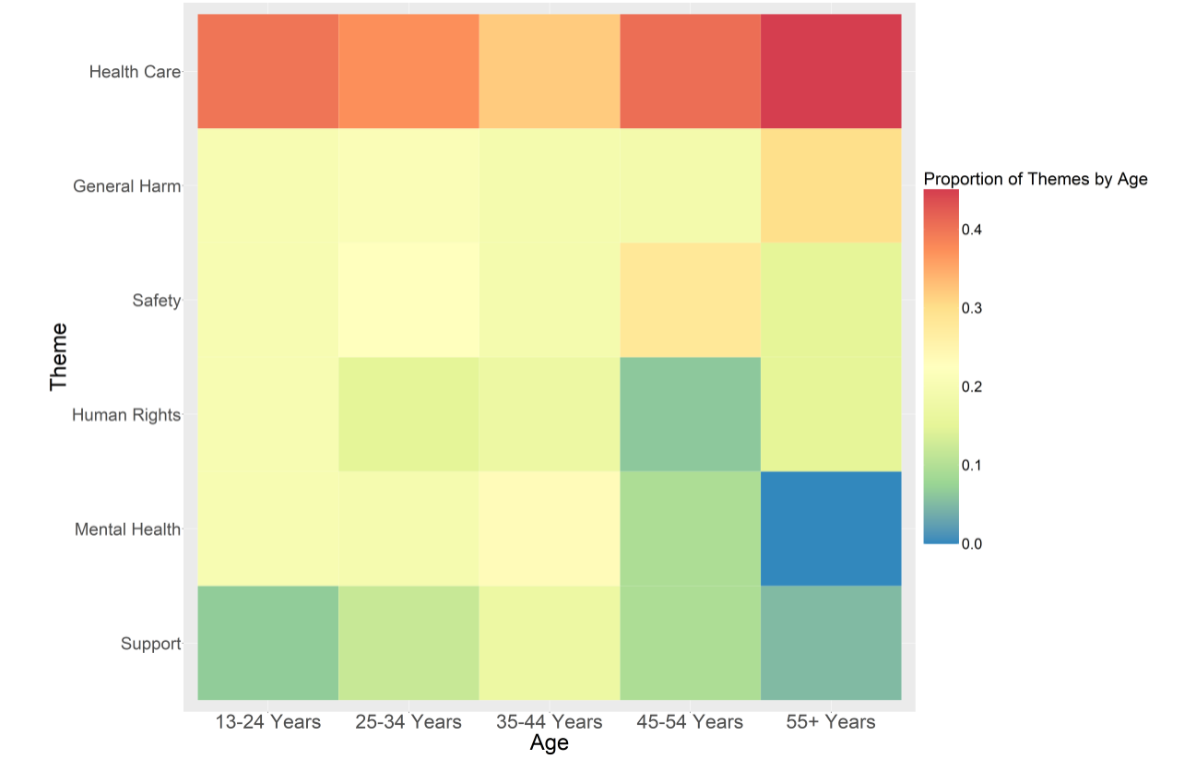
Health-related concerns of anti-LGBTQ+ (lesbian, gay, bisexual, transgender, queer/questioning) legislation expressed on Twitter by sexual minority men in the United States, by age (n=181).

ReportAGE [13] extracted the age for 188 (65.3%) of the 288 users, with 181 (62.8%) that were manually validated (Table 1). The timestamps that determined the users’ age with respect to the tweets in our thematic analysis showed that 15 (8.3%) users were aged 13-24 years, 67 (37%) were aged 25-34 years, 47 (26%) were aged 35-44 years, 32 (17.7%) were aged 45-54 years, and 20 (11%) were aged ≥55 years. *Health care* remained the top concern across all age groups (Figure 2). Although *safety* remained the next highest concern in the age groups of 25-34 years (22.4%) and 45-54 years (28.1%), it was less prevalent in the 35-44 years age group (19.1%) compared to *mental health* (23.4%). *Mental health*, however, was relatively low in the 45-54 years (9.4%) age group and not expressed in the ≥55 years age group (0%). Although *support* remained a relatively low concern across all age groups, it was more prevalent in the 35-44 years age group (17%).

## Discussion

### Principal Results

In this thematic analysis, *health care* was the top concern overall and across all regions and age groups. Our finding that *safety* was more of a concern in the Midwest and South than in the Northeast and West may reflect that many bills are being proposed in the Midwest and South, including 411 (77.5%) in 2024 [1]. Thus, concerns in the regions that would be more directly impacted by anti-LGBTQ+ legislation have focused more on specific and personal harms such as physical and life-threatening ones. Likewise, *mental health* was more prevalent than *general harm* in the Midwest and South, and *support* was more prevalent than *general harm* and *human rights* in the Midwest. In contrast, *human rights* was more prevalent than *mental health* and *support* in the West.

The variation of *mental health* across age groups may also reflect those who would be more directly impacted by anti-LGBTQ+ legislation—in particular, youth. Our finding that *mental health* was more prevalent among younger age groups supports findings that, among the general population, younger generations are more likely to report mental health concerns [16]. Although it may appear surprising that *support* was more prevalent in most of the older age groups, their tweets reveal many personal accounts reflecting on past experiences that raise concerns about the current generation of youth: “Had a friend text me about ‘Don’t say Gay’ bill and wanted my thoughts on it. Told him my experience as a kid and that I wish I had someone to talk to or resources about how I was feeling.”

### Strengths and Limitations

Our findings demonstrate that social media can be used to capture health-related concerns expressed by those who are directly impacted by anti-LGBTQ+ legislation. Based on our exploratory approach using thematic analysis, we found that *safety* was as much of a concern as *mental health* [2-5] overall and across the United States and most age groups. Our sample (n=288) was substantially larger than that in prior thematic analyses [6,7], addressed their limited distribution of age [6] and geographic location [7], and given that over 40% of the users were in the South, captured the voices of individuals who are least likely to discuss their sexual orientation in traditional settings [17]. Despite these strengths, our sample primarily included cisgender men and was collected solely from Twitter, which limits the generalizability of our results to the broader LGBTQ+ community and users of other social media platforms.

### Conclusions

We used social media for a thematic analysis of health-related concerns of anti-LGBTQ+ legislation among sexual minority men in the United States. Our findings may inform a broader range of health interventions and approaches for targeting them at specific populations of sexual minority men. By demonstrating that these concerns are expressed on social media, our findings can be leveraged by advocacy groups to amplify voices and rally public support for countering anti-LGBTQ+ bills. Directions for future work include incorporating data from more diverse LGBTQ+ identities and social media platforms.

## Appendix

Multimedia Appendix 1 Codebook.

## Abbreviations

LGBTQ+: lesbian, gay, bisexual, transgender, queer/questioning
WHO: World Health Organization

## Data Availability

The datasets analyzed during this study are available from the corresponding author on reasonable request.
