# Supplementary material for: Health-Related Concerns of Anti-LGBTQ+ Legislation: Thematic Analysis Using Social Media Data
Source: JMIR Infodemiology. 2025 Sep 11;5:e68956. doi: 10.2196/68956 (PMC12464498; doi:10.2196/68956)
Supplement: Multimedia Appendix 1 [file infodemiology_v5i1e68956_app1.docx]

**Identifying Tweets for a Thematic Analysis of Health-Related Concerns of Anti-LGBTQ+ Legislation: A Codebook**

This brief codebook will help coders identify tweets that express health-related concerns of anti-LGBTQ+ legislation for inclusion in a thematic analysis.

Tweets should be included if they refer to state-level anti-LGBTQ+ legislation and express a concern related to the World Health Organization’s broad definition of *health* as “a state of complete physical, mental and social well-being,” as in [1] and [2]:

1. Isn’t anyone going to talk about Missouri attempting to ban gender-affirming surgery and making it illegal to be a trans person?
2. It feels like checking a box, not a genuine reaction to the deadly anti-LGBTQ laws in Florida, Texas and other states.

While [1] and [2] explicitly refer to anti-LGBTQ+ legislation in Missouri, Florida, and Texas, [3] and [4] refer to specific state-level bills:

1. You haven’t made any comments/retweets about the inhumane #DontSayGay Bill
2. Does someone want to educate them on why this legislation is harmful and isn’t only limited to grade school, including the malicious intent behind the DON'T SAY GAY bill?

Because the tweets in this study were posted by individuals in the United States, tweets should also be included even if they do not explicitly refer to specific states or state-level bills, as in [5] and [6]:

1. Republicans are harming LBGT youth with their anti-LGBT, hateful policies and laws! And sadly, I’m not even surprised by this. According to NPR, “Nearly half of LGBTQ youth seriously considered suicide.”
2. LGBTQ+ folks are here to stay. The laws directed at us are simply acts of cruelty intended to increase homelessness, mental health crises, domestic abuse, and stigma.

However, tweets should not be included if they explicitly refer to federal-level or international anti-LGBTQ+ legislation, as in [7] and [8], respectively:

1. @realDonaldTrump… you take liberties away from trans people, so don’t pander for our votes. Your policies harm the LGBTQ community.
2. Hungary will hold a referendum on a new anti-#LGBTQ law, including questions such as "do you support holding sexual orientation workshops in schools without parental consent" and "should gender reassignment procedures be promoted among children"

In addition, tweets should not be included if they refer to equitable legislation or express health-related concerns that are not associated with legislation, as in [9] and [10], respectively:

1. Today, President Biden signed an executive order that includes an inclusive LGBTQ policy for schools, guidance for states to expand healthcare coverage for LGBTQ patients, an initiative to end conversion therapy, and more.
2. Thank goodness the @[username] spent all year rebranding with a fake physician title instead of condemning CRNA programs that BAN LGBT students. Patients should ask who’s involved in their anesthesia about their training, since we can call ourselves whatever we want apparently

While [11] and [12] refer to anti-LGBTQ+ legislation in Florida and South Carolina, they should not be included because they do not express health-related concerns:

1. I would love to see Walt Disney World and other major corporations pull out of Florida. Gay people should stop spending their tourist dollars in Florida. #DontSayGay is nothing but hate.
2. South Carolina joins about a dozen other states that have passed similar anti-trans laws in the last two years.
